# Intra-Rater and Inter-Rater Reliability of a Medical Record Abstraction Study on Transition of Care after Childhood Cancer

**DOI:** 10.1371/journal.pone.0124290

**Published:** 2015-05-22

**Authors:** Micòl E. Gianinazzi, Corina S. Rueegg, Karin Zimmerman, Claudia E. Kuehni, Gisela Michel, the Swiss Paediatric Oncology Group (SPOG)

**Affiliations:** 1 Department of Health Sciences and Health Policy, University of Lucerne, Lucerne, Switzerland; 2 Pediatric Hematology/Oncology, University Children’s Hospital, Bern, Switzerland; 3 Swiss Childhood Cancer Registry, Institute of Social and Preventive Medicine, University of Bern, Bern, Switzerland; Kyushu University Faculty of Medical Science, JAPAN

## Abstract

**Background:**

The abstraction of data from medical records is a widespread practice in epidemiological research. However, studies using this means of data collection rarely report reliability. Within the Transition after Childhood Cancer Study (TaCC) which is based on a medical record abstraction, we conducted a second independent abstraction of data with the aim to assess a) intra-rater reliability of one rater at two time points; b) the possible learning effects between these two time points compared to a gold-standard; and c) inter-rater reliability.

**Method:**

Within the TaCC study we conducted a systematic medical record abstraction in the 9 Swiss clinics with pediatric oncology wards. In a second phase we selected a subsample of medical records in 3 clinics to conduct a second independent abstraction. We then assessed intra-rater reliability at two time points, the learning effect over time (comparing each rater at two time-points with a gold-standard) and the inter-rater reliability of a selected number of variables. We calculated percentage agreement and Cohen’s kappa.

**Findings:**

For the assessment of the intra-rater reliability we included 154 records (80 for rater 1; 74 for rater 2). For the inter-rater reliability we could include 70 records. Intra-rater reliability was substantial to excellent (Cohen’s kappa 0-6-0.8) with an observed percentage agreement of 75%-95%. In all variables learning effects were observed. Inter-rater reliability was substantial to excellent (Cohen’s kappa 0.70-0.83) with high agreement ranging from 86% to 100%.

**Conclusions:**

Our study showed that data abstracted from medical records are reliable. Investigating intra-rater and inter-rater reliability can give confidence to draw conclusions from the abstracted data and increase data quality by minimizing systematic errors.

## Introduction

The abstraction of data from patients’ medical records (MR) is a widespread practice in epidemiological research, especially in retrospective studies [1–3]. Often, however, the reliability and internal validity of such data is questionable. This has several reasons. Firstly, data written in MR have not been produced for research purposes and the adequateness of such data for the study’s research question needs to be addressed [2]. Secondly, poor reliability due to the potential intra and inter-rater variance limits internal validity of the results. This is particularly true for multicenter studies in which several raters are involved, data collection conditions vary, MR formats differ, data come from different time periods and the data collection leaves room for interpretation [1]. For this reasons it is important to report reliability of such studies. Further, this can help to improve the collection process, to reduce and correct problems or discrepancies, and, later, to gain confidence in the conclusions that will be drawn [4]. Despite the importance of reporting such measures, only few studies actually do so [2,4–8]. In general, published retrospective chart reviews which assessed reliability report good reliability levels for their abstracted data, but we have to remember that publication bias might be a problem in this type of study with only studies with positive results being published.

The project “Transition after Childhood Cancer (TaCC)” aims to assess the transition from pediatric to adult care of childhood cancer survivors in Switzerland by collecting data from MR in nine clinics and in three language regions. Because no previous study assessed transition using a systematic chart review for data collection we had to develop and pilot an abstraction form based on available literature and project aims. For these reasons we found it important to assess the reliability of collected data by investigating a) intra-rater reliability of two raters at two time points; b) the possible learning effects over time comparing each rater to a gold-standard at two time points; and c) inter-rater reliability.

## Methods

### Ethics statement

Ethics approval was provided through the general cancer registry permission of the Swiss Childhood Cancer Registry (The Swiss Federal Commission of Experts for Professional Secrecy in Medical Research) and a non obstat statement was obtained from the ethics committee of the canton of Bern, stating that no additional ethics permission and no additional informed consent was necessary. All information regarding individuals was made anonymous to investigators prior to analysis.

### Study population

The «Transition after Childhood Cancer (TaCC) » study is a retrospective multicenter study conducted within the population-based Swiss Childhood Cancer Registry (SCCR). For nearly four decades the SCCR has been collecting data on all patients diagnosed with leukemia, lymphoma, central nervous system (CNS) tumors, malignant solid tumors or Langerhans cell histiocytosis before the age of 21 years [9,10]. The TaCC study included a stratified (by diagnosis and treating clinic) randomly selected sample of patients registered in the SCCR, who were diagnosed with childhood cancer at an age between 0 and 15 years, who survived ≥ 5 years and were aged ≥16 years at the time of this study.

### Initial data collection for the TaCC study

Within the TaCC study we conducted a systematic MR abstraction at the 9 clinics with pediatric oncology wards throughout Switzerland (all clinics were affiliated to the Swiss Paediatric Oncology Group). Data collection started in March 2012 and ended in April 2013. For data collection we utilized a standardized abstraction form on hardcopy, which we developed using the available literature on chart reviews [11]. As suggested by the guidelines, we piloted the abstraction form in three of the nine clinics, before the actual data abstraction started. We collected data on the following main categories: frequency of follow-up visits after the age 16 years, medical professionals involved, discharge (patient discharged from pediatric oncology without being transferred), date of discharge, discharge planned, date of planned discharge, transfer (patient transferred from pediatric oncology to an adult medical professional), transfer destination, date of transfer, missed follow-up appointments (the patient did not go to a visit). We digitally photographed all relevant documents as “back up” and saved them on secure servers. Following data collection, we used Epidata 3.1 to enter our data into a database. All baseline demographic or clinical information were directly extracted from the SCCR database.

### Sample for reliability assessment

The number of re-abstractions we carried out was based on the number of medical charts available containing information about the variables to be extracted as well as on formal sample size calculations for the kappa statistic [12]. Using alpha and beta error rates of 0.05 and 0.2, respectively, when testing for a statistical difference between moderate (i.e., 0.40) and high (i.e., 0.75) kappa values, sample size estimates ranged from 77 to 28 when the trait prevalence was varied between 10% and 50%. Thus, our sample sizes for intra-rater reliability and inter-rater provided the needed power to detect differences. We selected all medical records that did not have any missing values in the variables under investigation. We conducted the re-abstraction in the first three clinics of the same language region.

### Re-abstraction

For the re-abstraction we focused exclusively on the most important variables, namely: the variables “still in pediatric follow-up (yes, no)”, “transferred (yes, no)”, “discharged (yes, no)”, “transfer destination” (general practitioner, adult oncologist, other specialist), and the date variables “date of transfer”, “date of discharge” and “date of next visit in pediatric oncology” (Table 1).

**Table 1:** Variables assessed in the re-abstraction.

Variable	Description	Categories
**Transfer**	This variable is used to assess whether the patient has been transferred from pediatric oncology to an *adult* medical professional.	Yes/No
**Discharge**	This variable is used to assess whether the patient has been discharged from pediatric oncology without being referred to another medical professional.	Yes/No
**In Follow-up**	This variable is used to assess whether the patient had regular follow-up visits in pediatric oncology at the time of data collection	Yes/No
**Transfer destination**	This variable is used to assess to which adult health professional the patient has been transferred to.	1.General Practitioner 2. Adult Oncologist 3. Other Specialist
**Transfer date**	Here the day, month and year of transfer had to be indicated.	dd, mm, yyyy
**Discharge date**	Here the day, month and year of discharge had to be indicated.	dd, mm, yyyy
**Date of next visit in pediatric oncology**	Here the day, month and year of the next visit at pediatric oncology have to be indicated.	dd, mm, yyyy

The categorical variables represent a more challenging collection than date variables because the corresponding information had to be found in free text and often necessitated an interpretation.

### Medical record *raters*

Three study raters were chosen to carry out the abstraction in the different clinics based on their linguistic knowledge (they had to be proficient in all national languages) and on their level of education. All raters held a degree at the Master level, one in pedagogy/psychology, the second in social sciences and the third in biology. None of the raters had clinical experience, which we believed was not necessary for the purpose of this abstraction. One of these three raters (Master in Biology) had joined the research team later and was therefore excluded from the reliability study.

Both raters included in the reliability study were trained prior to data collection for the most important concepts assessed in the TaCC study. To measure intra-rater reliability the 2 raters abstracted a selected sample of medical records at two points in time. Both raters did not have access to the results collected at time 1.

To investigate possible learning effects between time point 1 and time point 2, the project manager (MEG) also abstracted the data of the same patients. These data were considered the gold standard and results of the two raters at time point 1 and 2 were than compared against the gold standard. To assess inter-rater reliability the 2 raters independently abstracted data of the same study subjects at time point 2.

### Statistical analysis

We performed all analyses using Stata 12.0 (StataCorp, College Station, TX). We first calculated percentage agreement, i.e. the proportion of assessments in which the two observations agreed, Cohen’s kappa and Prevalence-Adjusted Bias-Adjusted Kappa (PABAK) for all variable in intra-rater and inter-rater comparison [13]. For the intra-rater reliability analysis we present results per rater when possible. To assess possible learning effects between point in time 1 and point in time 2, we calculated the Cohen’s kappa between data collected by each rater at the two points in time and the data collected by the project manager MEG (gold standard).

### Cohen’s Kappa and Adjusted Kappa

Kappa indicates a numeric rating of the degree of agreement between two raters, taking into account the degree of agreement which would be expected by chance. The calculation of Cohen’s kappa is based on the difference between the agreement that is actually present (Pr_a_) and the agreement obtained by chance alone (Pr_e_) (Formula 1) [14]. Kappa’s values range from 0 to 1 with 0 meaning “less than chance agreement” and 1 “almost perfect agreement”.

$$k=\frac{\text{Pr}\left(a\right)-\text{Pr}\left(e\right)}{1-\text{Pr}\left(e\right)}$$ (Formula 1)

Formula 1, however, does not take into account the bias between observers (the extent of disagreement) or the distribution of data across the categories that are used (prevalence). The following example will show how identical agreement can lead to different coefficients of kappa because of the different prevalence of data across the categories.

In both Tables 2 and 3 there is equal agreement (60 from yes and no: 25+35 and 45+ 15). However, if we apply Formula 1 to calculate Cohen’s kappa we will end up with different results (K_1_ = 0.1304 and K_2_ = 0.2593). This difference in results occurs because of the different distribution of data in the 2x2 cells (the so called prevalence) [12].

**Table 2:** Kappa example 1.

Rater 2	Rater 1		
	Yes	No
Yes	45	15
No	25	15

**Table 3:** Kappa example 2.

Rater 2	Rater 1		
	Yes	No
Yes	25	35
No	5	35

The interpretation of kappa alone without any indication of prevalence or bias can be imprecise. To overcome this problem an alternative form of kappa has been proposed which takes into account both bias and prevalence [15]. This is summarized by the Prevalence-Adjusted Bias-Adjusted Kappa (PABAK). PABAK gives the proportion of agreement beyond expected chance agreement regardless of unbalanced data patterns. The interpretation of PABAK is the same as for kappa. If we consider a 2x2 table like the one in Table 4, PABAK is calculated as in Formula 2.

**Table 4:** Example for the Prevalence-Adjusted Bias-Adjusted Kappa (PABAK).

Rater 2	Rater 1		
	Yes	No
Yes	a	b
No	c	d

$$PABAK=\frac{\left(a+b\right)-\left(b+c\right)}{n}$$ (Formula 2)

### Interpretation of Cohen’s kappa

To interpret our result we used as benchmark the cut-off proposed by Landis and Koch [16] according to whom Cohen’s kappas ≥ 0.80 represent excellent agreement, coefficients between 0.61 and 0.80 represent substantial agreement, coefficients between 0.41 and 0.61 moderate agreement and <0.41 fair to poor agreement.

## Results

### Sample

**Fig 1:**
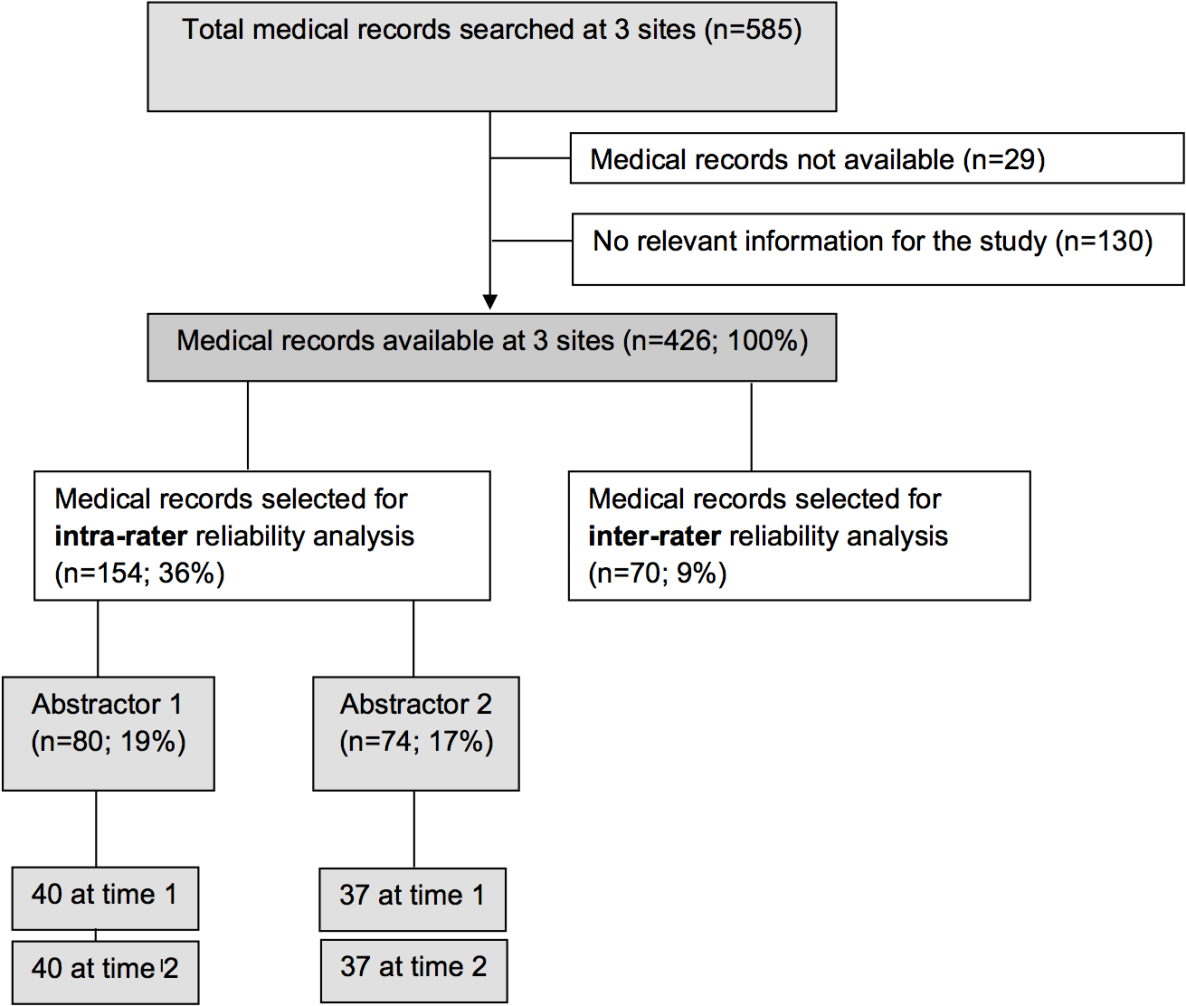
Flow chart of sample selection for reliability assessment. Fig 1 shows the flow chart of our study population starting from those eligible to those included in the analysis.

The final analysis included 154 records for the assessment of intra-rater reliability. Of these, 80 had been viewed by rater 1 and 74 by rater 2. Mean time between first (point in time 1) and second abstraction (point in time 2) was 7.6 months (SD = 2.2), range (2.1–10.3 months). For the assessment of inter-rater reliability we included 70 records (Fig 1).

### Intra-rater reliability

**Fig 2:**
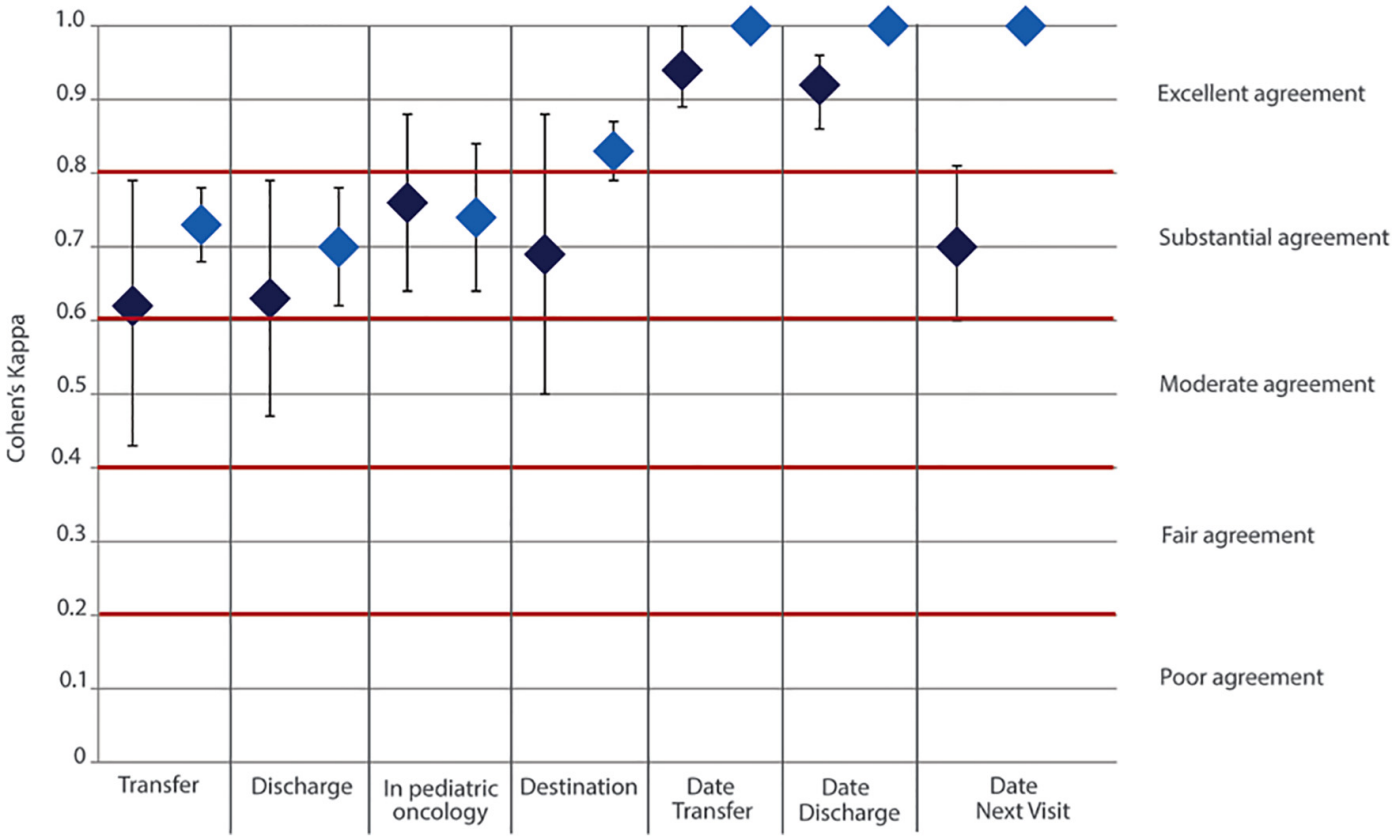
Kappa values and their interpretation for intra-rater and inter-rater reliability. Fig 2 shows the values of kappa for intra-rater (dark blue) and for inter-rater (light blue) reliability with their confidence intervals T for each variable under investigation.

Overall, all variables assessed had substantial (Cohen’s kappa ≥ 0.6) to excellent agreement (Cohen’s kappa ≥ 0.8) with an observed percentage agreement ranging from 75% (date of next visit in pediatric oncology) to 95% (date of transfer) (Fig 2).

**Fig 3:**
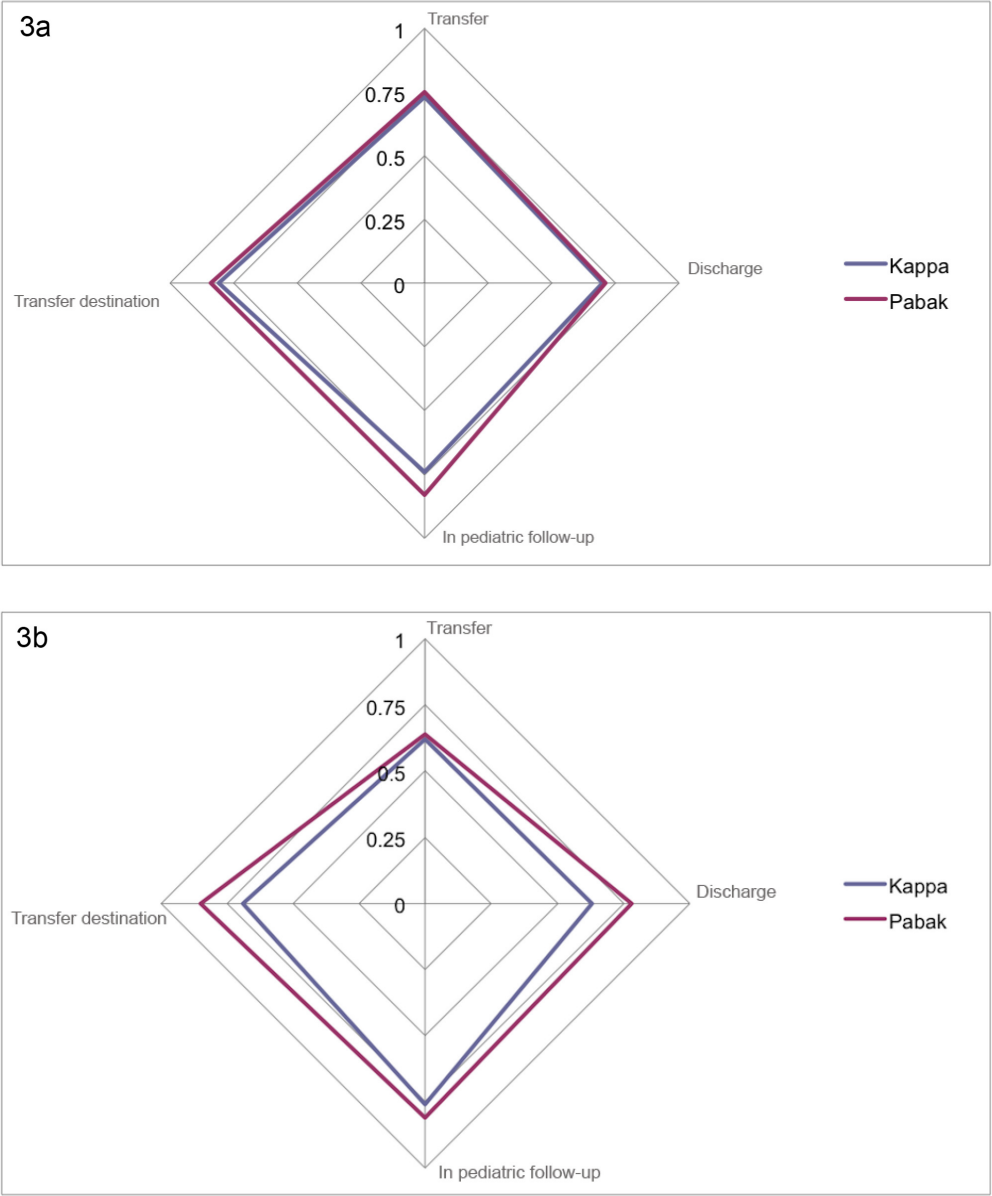
Kappa values and Prevalence-adjusted Bias-adjusted kappa values for intra-rater (a) and inter-rater reliability (b). Fig 3a and 3b show the values of kappa compared to the the values obtained by calculating the Prevalence-adjusted Bias-adjusted kappa for intra-rater reliability (a) and inter-rater reliability (b).

After taking into account prevalence and bias, PABAK was higher for all variables and ranged from 0.64 to 0.81 than the unadjusted kappa values (Table 5; Fig 3).

**Table 5:** Intra-rater reliability.

Variable	Agreement	kappa	95% CI	PABAK
**Transfer**	**82%**	**0.62**	**0.43–0.79**	**0.64**
*Rater 1*	*85%*	*0*.*67*	*0*.*43–0*.*91*	*0*.*73*
*Rater 2*	*77%*	*0*.*48*	*0*.*20–0*.*73*	*0*.*58*
**Discharge**	**84%**	**0.63**	**0.47–0.79**	**0.78**
*Rater 1*	*90%*	*0*.*77*	*0*.*57–0*.*98*	*0*.*79*
*Rater 2*	*75%*	*0*.*45*	*0*.*22–0*.*75*	*0*.*47*
**In Follow-up**	**88%**	**0.76**	**0.51–0.87**	**0.81**
*Rater 1*	*90%*	*0*.*77*	*0*.*64–0*.*91*	*0*.*80*
*Rater 2*	*86%*	*0*.*58*	*0*.*49–0*.*67*	*0*.*71*
**Transfer destination**	**88%**	**0.69**	**0.64–0.88**	n.a.^d^
*Rater 1*	*91%*	*0*.*74*	*0*.*62–0*.*86*	n.a.^d^
*Rater 2*	*82%*	*0*.*51*	*0*.*40–0*.*62*	n.a.^d^
**Date of transfer**	**95%**	**0.94**	**0.89–1.00**	**n.a.** ^**d**^
*Rater 1*	*91%*	*0*.*89*	*0*.*74–1*.*00*	*n*.*a*.
*Rater 2*	*100%*	1.00	*-*	n.a.
**Date of discharge**	**93%**	**0.93**	**0.81–0.95**	**n.a.** ^**d**^
*Rater 1*	*100%*	*1*.*00*	*-*	*n*.*a*.^*d*^
*Rater 2*	*86%*	0.85	*0*.*66–0*.*91*	*n*.*a*.^*d*^
**Date of next visit**	**75%**	**0.70**	**0.50–0.82**	**n.a.** ^**d**^
*Rater 1*	*78%*	*0*.*70*	*0*.*45–0*.*82*	*n*.*a*.^*d*^
*Rater 2*	*67%*	*0*.*50*	*0*.*40–0*.*81*	*n*.*a*.^*d*^

Abbreviations: CI, Confidence Interval; kappa, Cohen’s kappa; n.a., not applicable; PABAK, Prevalence and Bias Adjusted Kappa.

The variable “in follow-up” had the highest Cohen’s kappa (k = 0.76), while transfer and discharge had the lowest (k = 0.62 and k = 0.63) (Fig 2).

Date variables had Cohen’s kappas (k) above 0.9 except “date of next visit” were k was 0.7.

When looking at the results stratified by the two raters, we could see that Cohen’s kappas were consistently higher for rater 1 than for rater 2. Especially for the variables transfer and discharge rater 2 had Cohen’s kappas < 0.5 (Table 5).

### Learning effects

**Fig 4:**
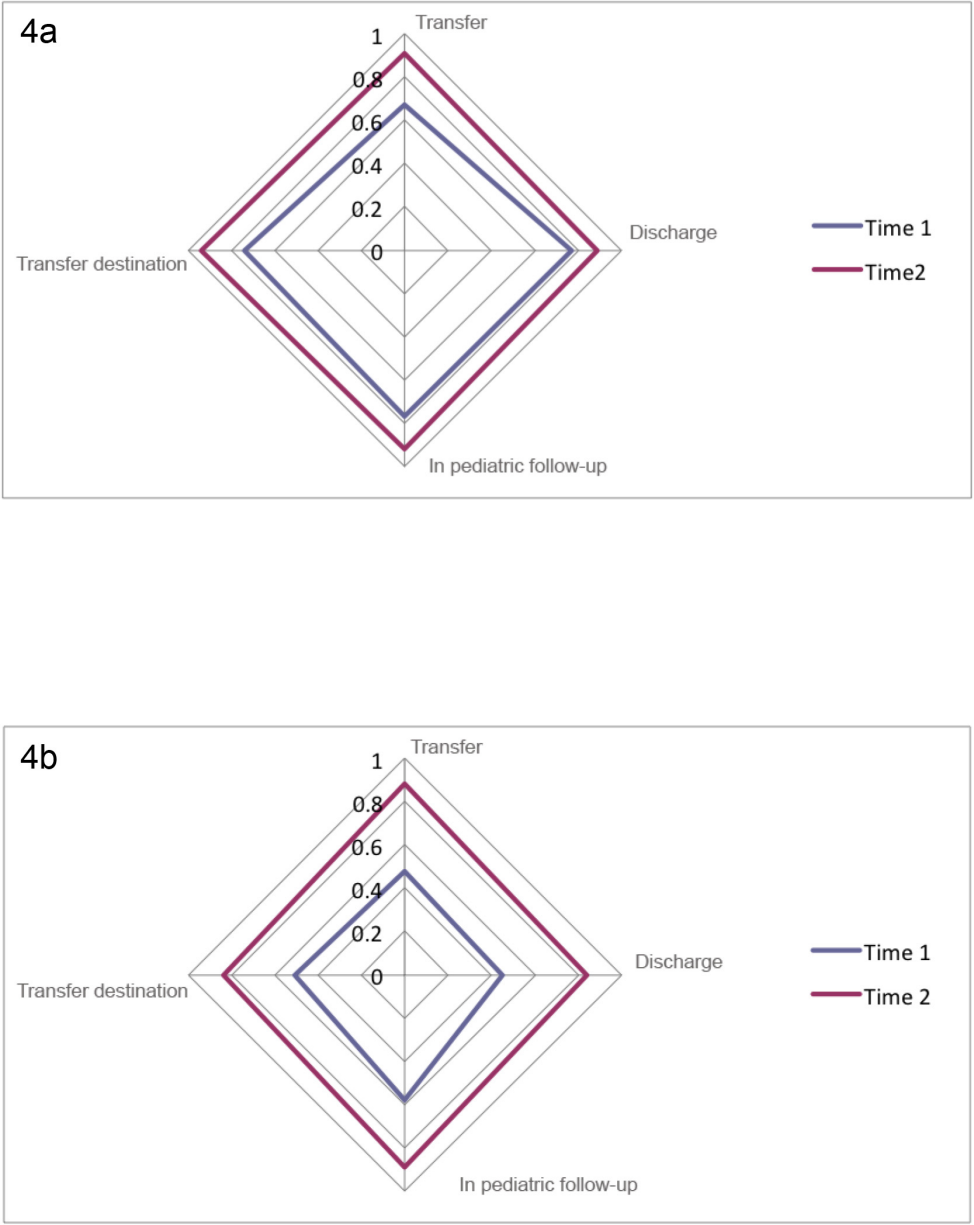
Learning effect of the two raters at two points in time compared with the abstraction of the project manager. Fig 4a and 4b show the comparison of the abstraction at two points in time of rater 1 (a) and rater 2 (b) compared to the chosen golden standard (abstraction of the project manager).

When looking at learning effects between point in time 1 and point in time 2 against the data collected by the project manager MEG we can see that kappa’s values greatly improved for both raters (all p_s_ <0.001) (Fig 4).

### Inter-rater reliability

For the variables transfer, discharge, in follow up and transfer destination the observed agreement was high ranging from 86% to 91% (Table 6).

**Table 6:** Inter-rater reliability.

Variable	Agreement	kappa	95% CI	PABAK
**Transfer**	88%	**0.73**	0.68–0.78	0.75
**Discharge**	86%	**0.70**	0.62–0.78	0.71
**In Follow-up**	91%	**0.74**	0.64–0.84	0.81
**Transfer destination**	89%	**0.83**	0.71–0.88	n.a.^d^
**Date of transfer**	100%	**1.00**	-	n.a.^d^
**Date of discharge**	100%	**1.00**	-	n.a.^d^
**Date of next visit**	100%	**1.00**	-	n.a.^d^

Abbreviations: CI, Confidence Interval; kappa, Cohen’s kappa; n.a., not applicable; PABAK, Prevalence and Bias Adjusted Kappa.

Cohen’s kappas reached substantial or excellent agreement ranging from 0.70 (discharge) to 0.83 (transfer destination) (Table 6; Fig 2). After adjusting for prevalence and bias, the true proportion of agreement (PABAK) was higher for all variables and ranged from 0.71 to 0.84 (Table 6). Among the categorical variables, “in follow-up” had the highest Cohen’s kappa (k = 0.76), while transfer and discharge had the lowest (k = 0.62 and k = 0.63). Agreement and kappa were perfect (100%; k = 1) for the three date variables assessed.

## Discussion

Results of our study showed that for both intra-rater and inter-rater we had substantial to excellent agreement. As expected, the variables for which no interpretation was necessary (e.g. date variables), higher and often perfect agreement was present. We found that one rater consistently had lower intra-rater agreement, but further analysis showed an improvement of judgment between point in time 1 and point in time 2 for both raters when compared to the chosen gold standard. Unexpectedly, Cohen’s kappas were higher for inter-rater reliability than for intra-rater reliability.

### Strengths and Limitations

This is the first study assessing transition from pediatric to adult care with medical records which tested for intra-rater and inter-rater reliability of the collected data. Because the variables assessed were not always easy to find in the medical records nor easy to rate, these results give us the confidence needed to interpret the data collected. Because data collection was still ongoing, assessing reliability also gave us the opportunity to identify possible problems related to the rater’s comprehension and intervene in case we had the impression systematic errors were occurring. For data which we had already collected we performed a double control to make sure the possible mistakes of the first phase could be corrected.

The study has however limitations. Firstly, the sample size did not allow detection of inter-hospital differences or differences between different types of medical records (i.e. paper versos micro film) or archiving periods, which could possibly explain some of the rater variability. We also included three clinics only, while the whole study was carried out in a total of nine, in three different language regions. Caution is therefore needed in the generalization of results. In our study we only looked at documents from pediatric oncology and, even though they contain correspondence with the other specialists involved in follow-up, it was often difficult to fully understand the patients’ medical history. This was further aggravated by the fact, that none of the raters had expertise in clinical practice and was familiar with the local documentation systems. Finally, kappa has known limitations which we tried to overcome by reporting the prevalence-adjusted bias-adjusted kappa as proposed by several authors [12,15].

### Comparison with other studies

None of the studies that looked at assessing transition from pediatric to adult care did investigate reliability of the abstracted data. We found several studies assessing mostly inter-rater reliability of diagnostic tests (screening and detection of adverse events) which were not directly comparable to ours. Two studies [4, 8] were more similar in methodology and scope to ours: in the first one the authors found that agreement was poorer for variables for which a degree of interpretability was needed (judgment data), while it was higher for data such as demographic or numeric characteristics [8]. The same was found in the second multicenter study using medical record abstraction in a study on community-based asthma care program [4]. In this study they found that the multicenter abstraction of data from medical records is reliable and conclusions could be drawn from the results. They found an overall Cohen’s kappa for intra-rater reliability of 0.81 (excellent) and an overall Cohen’s kappa of 0.75 (substantial) in the inter-rater analysis.

### Interpretation of results

Even though we could not reach perfection in the abstraction of the data, our results are reassuring and showed satisfactory levels of agreement. Further, the raters’ improvement in judgment between time 1 and time 2, probably due to a learning effect, allows to assume that the abstraction in the remaining 6 clinics not included in the present study, is of at least similar or higher quality and reliability. As it was expected, agreement was higher for non-judgment variables such as dates. Such information mostly does not require interpretation. Other data such as the variables “transfer” or “transfer destination” were to be looked for in free texts such as letters or medical reports and it often required a different degree of attention and interpretation. Indeed, data abstraction was difficult because in the pediatric oncology setting several other specialists are often involved in the follow-up of patients (e.g. neurologists, endocrinologists). Documents found were often from various specialists and the raters had to decide whether, for example, a patient was transferred from pediatric oncology or whether the patient was actually transferred from another specialist. A patient could namely still be in follow-up in pediatric oncology, but might have been transferred from pediatric endocrinology to adult endocrinology. This was often a source of confusion when abstracting data.

In contrast to other studies [4,7], we found higher inter-rater reliability than intra-rater. Because inter-rater reliability was assessed at point in time 2 only this higher reliability may be due to the learning effect we could show.

### Implications for practice

Despite the well-known limitations of retrospective studies using MR or other secondary data, an increasing number of studies have shown that such an approach can produce reliable results if the procedure is consistent and standardized, and if raters are appropriately trained. It would be interesting to investigate whether the archives’ organization, the documents’ age and the format of such documents (e.g. microfilm, electronic, hardcopy) influence the quality of the retrieved data. Finally, such an analysis could help detect possible problems such as rater’s comprehension difficulties or discrepancies and improve the overall quality of retrospective studies.

## Conclusion

Our study showed that despite several limitations attributed to data abstracted from MR, our data seems to be reliable. Thanks to the assessment of learning effects, systematic errors could be corrected and general data quality improved. With good training and a standardized procedure good reliability can be achieved.
